# Supplementary material for: ABHD17 proteins are novel protein depalmitoylases that regulate N-Ras palmitate turnover and subcellular localization
Source: eLife. 2015 Dec 23;4:e11306. doi: 10.7554/eLife.11306 (PMC4755737; doi:10.7554/eLife.11306)
Supplement: Supplementary File 1. — A summary table compiling the 29 serine hydrolases targeted by HDFP (>25% activity inhibition) as determined by cABPP-SILAC (Stable isotope labeling of amino acids in culture) in (Martin et al., 2011). LYPLAL1 (APT1L) was added to this list as a candidate enzyme for Palmostatin B testing (Tian et al., 2012). DOI: http://dx.doi.org/10.7554/eLife.11306.012 [file elife-11306-supp1.docx]

**Supplementary file 1.** List of Metabolic serine hydrolases inhibited by HDFP.

|  | **Enzyme** | **Alias** | **HDFP%*** | **GENE ID** | | **M. Wt.** | | **Source (Cloning template)** | | **CLONING INFO (TABLE S2)** | |  |
| --- | --- | --- | --- | --- | --- | --- | --- | --- | --- | --- | --- | --- |
| 1 | **LYPLA1** | **APT1** | **95** | **10434** | | 24kDa | | pMyc-hAPT1 (Dr. Takashi Izumi) | |  | |  |
| 2 | **LYPLA2** | **APT2** | **95** | **11313** | | 25kDa | | Dharmacon (MHS6278-202756028) | | **#1. FLAG-APT2** | |  |
| 3 | **PPT1** | **CLN1** | **95** | **5538** | | 37kDa | |  | |  | |  |
| 4 | **PNPLA6** | **NTE** | **95** | **10908** | | ~150kDa | | Dharmacon (MHS1010-202802613) | | **#2. FLAG-PNPLA6** | |  |
| 5 | **PNPLA7** | **NTEL1** | **95** | **375775** | | ~148kDa | |  | |  | |  |
| 6 | **PNPLA8** | **iPLA2γ** | **95** | **50640** | | 63kDa | |  | |  | |  |
| 7 | **PGAP1** | **-** | **95** | **80055** | | ~85kDa | |  | |  | |  |
| 8 | **PAFAH2** | **-** | **95** | **5051** | | ~40kDa | | Dharmacon (MHS6278-202827903) | | **#3. FLAG-PAFAH2** | |  |
| 9 | **FAAH** | **-** | **95** | **2166** | | ~60kDa | | Dharmacon (MHS6278-202802173) | | **#4. FLAG-FAAH** | |  |
| 10 | **FASN** | **FAS** | **94** | **2194** | | 270kDa | | Endogenous | |  | |  |
| 11 | **LIPE** | **HSL** | **91** | **3991** | | 84kDa | |  | |  | |  |
| 12 | **ABHD6** | **-** | **92** | **57406** | | ~30kDa | | Dharmacon (MHS6278-202826707) | | **#5. FLAG-ABHD6** | |  |
| 13 | **ABHD10** | **-** | **85** | **55347** | | 34kDa | | hORFeome | | **#6. ABHD10 in pCINeo** | |  |
| 14 | **ABHD12** | **PHARC** | **95** | **26090** | | ~45kDa | |  | |  | |  |
| 15 | **ABHD13** | **C13orf6** | **95** | **84945** | | 39kDa | |  | |  | |  |
| 16 | **AADACL1** | **NCEH1** | **94** | **57552** | | ~50kDa | |  | |  | |  |
| 17 | **FAM108A1** | **ABHD17A** | **94** | **81926** | | 39kDa | | hORFeome | | **#7. ABHD17A-FLAG** | |  |
| 18 | **FAM108B1** | **ABHD17B** | **94** | **51104** | | 32kDa | | Dharmacon (MHS1010-202726047) | | **#8. ABHD17B-FLAG** | |  |
| 19 | **FAM108C1** | **ABHD17C** | **94** | **58489** | | 36kDa | | Dharmacon (MHS6278-202806242) | | **#9. ABHD17C-FLAG** | |  |
| 20 | **BAT5** | **ABHD16A** | **95** | **7920** | | 63kDa | | hORFeome | | **#10. FLAG-ABHD16A** | |  |
| 21 | **DPP8** | **-** | **90** | **54878** | | ~100kDa | |  | |  | |  |
| 22 | **ABHD4** | **-** | **70** | **63874** | | 40kDa | | hORFeome | | **#11. FLAG-ABHD4** | |  |
| 23 | **LIPA** | **-** | **54** | **3988** | | ~40kDa | |  | |  | |  |
| 24 | **ACOT1** | **-** | **52** | **641371** | | 43kDa | | hORFeome | | **#12. ACOT1-HA** | |  |
| 25 | **ACOT2** | **-** | **50** | **10965** | | 45kDa | | DNASU (HsCD00045505) | | **#13. ACOT2-HA** | |  |
| 26 | **PREPL** | **-** | **52** | **9581** | | ~80kDa | |  | |  | |  |
| 27 | **SERHL2^†^** | **-** | **35** | **253190** | | ~36kDa | |  | |  | |  |
| 28 | **LYPLA3** | **PLA2G15** | **35** | **23659** | | ~40kDa | |  | |  | |  |
| 29 | **PARL** | **PSARL** | **28** | **55486** | | ~43kDa | |  | |  | |  |
| 30 | **LYPLAL1^‡^** | **APT1L** | **N/A** | **127018** | | 24kDa | | hORFeome | | **#14. APT1L in pCINeo** | |  |
|  |  |  |  |  | |  | |  | | |  | |
| **Excluded from this study: GREEN- Established luminal activity; RED- Established proteases; PURPLE- Not included in ABPP analyses.** | | | | | | | | | | | | |
| ***** As reported in (Martin et al., 2011). | | | | |  | |  | |  | |  | |
| **^†^** Homologue of mouse SERHL.  **^‡^** Was absent from (Martin et al., 2011). | | | | |  |  |  |  |  |  |  |  |
