# Supplementary material for: ABHD17 proteins are novel protein depalmitoylases that regulate N-Ras palmitate turnover and subcellular localization
Source: eLife. 2015 Dec 23;4:e11306. doi: 10.7554/eLife.11306 (PMC4755737; doi:10.7554/eLife.11306)
Supplement: Supplementary File 2. — A table listing PCR primers used to subclone candidate serine hydrolases for cABPP, pulse-chase/click chemistry, and confocal imaging studies. DOI: http://dx.doi.org/10.7554/eLife.11306.013 [file elife-11306-supp2.docx]

**Supplementary file 2.** List of cloning oligos used in this study.

| **#** | **Plasmid** | **Oligo Pair Sequences** |
| --- | --- | --- |
| **1** | **FLAG-APT2**  **(in NT-FLAG)** | 5'-ATTTAAGAATTCATGTGTGGTAACACCATGTCTGTG-3'  5'-ATTTATCTCGAGTTAGACAGGAGGCAGCAGC-3' |
| **2** | **FLAG-PNPLA6**  **(in NT-FLAG)** | 5'-ATTACGAATTCATGGAGGCTCCACTGCAAAC-3'  5'-TCATGCTAGCTTATGCATCTGTGGCTGAGCC-3' |
| **3** | **FLAG-PAFAH2**  **(in NT-FLAG)** | 5'-AATACGAATTCATGGGAGTCAACCAGTCTGTG-3'  5'-ACTTCTCGAGTTACAGGCTGGACAGATGGTG-3' |
| **4** | **FLAG-FAAH**  **(in NT-FLAG)** | 5'-AATACGAATTCATGGTGCAGTACGAGCTGTG-3'  5'-ACTTCTCGAGTTAGGATGACTGCTTTTCAGGGG-3' |
| **5** | **FLAG-ABHD6**  **(in NT-FLAG)** | 5'-AATACGAATTCATGGATCTTGATGTGGTTAACATGTTTG-3'  5'-ATAACTCGAGTCAGTCCAGCTTCTTGTTGTTGTC-3' |
| **6** | **ABHD10**  **(in pCINeo)** | 5'-ATATCAGAATTCATGGCTGTTGCGCGC-3'  5'-TATCCTCTCGAGCTAGTTCACGATAGTTGAGAGCTTATCAATTAAG-3' |
| **7** | **ABHD17A-FLAG**  **(in pCINeo)** | 5'-ATATCAGAATTCATGAATGGGCTGTCGCTGAGTG-3'  5'-ATAGCCTCGAGCTATTTATCGTCATCGTCTTTGTAATCTGCACGCTGGC TGGG-3' |
| **8** | **ABHD17B-FLAG**  **(in pCINeo)** | 5'-TTTACGAATTCATGAACAATCTTTCATTTAGTGAGCTATG-3'  5'-ATAGCGTCGACTTATTTATCGTCATCGTCTTTGTAATCCAAATTTACC  AGTTCCTGTGACAC-3' |
| **9** | **ABHD17C-FLAG**  **(in pCINeo)** | 5'-AATACGAATTCATGAACGGCTTCTCGCTGG-3'  5'-ATAGCGTCGACTCATTTATCGTCATCGTCTTTGTAATCGGAGTTAGG  AAGTTCGTGAG-3' |
| **10** | **FLAG-ABHD16A**  **(in NT-FLAG)** | 5'-ATATCAGAATTCATGGCAAAGCTGCTGAGC-3'  5'-ATAGCTTCTAGACTAGAGGTGCCAGGGCATC-3' |
| **11** | **FLAG-ABHD4**  **(in NT-FLAG)** | 5'-ATATCACAATTGATGGCCGATGATCTGGAGC-3'  5'-TATGCTCTCGAGTCAATCAACTGAGTCGCAGATCTCC-3' |
| **12** | **ACOT1-HA**  **(in pCINeo)** | 5'-ATATCAGAATTCATGGCTGCGACGCTG-3'  5'-TATGCTCTCGAGTTACACTTTTGATGGGATTGTCCCCTC-3' |
| **13** | **ACOT2-HA**  **(in pCINeo)** | 5'-ATCTCACAATTGATGTCTAACAAGCTTCTTTCTCCCCAC-3'  5'-TATGCTCTCGAGTTACACTTTTGATGGGATTGTCCCCTC-3' |
| **14** | **APT1L**  **(in pCINeo)** | 5'-ATATCAGAATTCATGGCGGCTGCGTCG-3'  5'-ATAGCTCTCGAGTCATTTTTGTTTTTCCATTTCTCCTGGCAGC-3' |
| **15** | **ABHD17A-S211A**  **-FLAG**  **(in pCINeo)** | 5’-GCATCATCCTGTACGGTCAGGCAATTGGCACGGTGCCCACCG-3’ (F)  5’-CGGTGGGCACCGTGCCAATTGCCTGACCGTACAGGATGATGC-3’ |
| **16** | **ABHD17A ΔN**  **-FLAG**  **(in pCINeo)** | 5’-ATATCAGAATTCATGCGCATCGCTGCCAAGC-3’ (F)  5'-ATAGCCTCGAGCTATTTATCGTCATCGTCTTTGTAATCTGCACGCTG  GCTGGG-3' |
| **17** | **ABHD17A-mCherry-Linker** | 5’-GGTGGCGACCGGTGGATCCTTTGCACGCTGGCTGGG-3’ (Reverse only)  ** Reverse oligo paired with 15(F) and 16(F) to generate ABHD17A1 fragments.* |
| **18** | **C-terminal mCherry Cassette for ABHD17A** | 5’-CAAAGGATCCACCGGTCGCCACCATGGTGAGCAAGGGCGAGG-3’  5’-TATAGCTAGCTCTAGATTACTTGTACAGCTCGTCCATGCCG-3’ |
| **19** | **N-terminal mCherry Cassette for**  **APT1 & APT2** | 5’-ATAATGAATTCCAATTGCCGCCACCATGGTGAGCAAGGGCG-3’  5’-CAGTATTCGAAGCTTGAGCTCGAGATCTGAGTACTTGTACAGCTCGT  CCATGC-3’ |
| **20** | **mCherry-Linker-APT1** | 5'-TACTCAGATCTCGAGCTCAAGCTTCGAATACTGATGTGCGGCAATAA  CATGTCAAC-3’  5’- ATCCCGGGTTCTAGAAACTCAATCAATTGGAGGTAGGAGTTTATCAATG-3’ |
| **21** | **mCherry-Linker-APT2** | 5’- TACTCAGATCTCGAGCTCAAGCTTCGAATACTGATGTGTGGTAACA  CCATGTCTGTG-3’  5’- ATCCCGGGTTCTAGAAACTTAGACAGGAGGCAGCAGC-3’ |
