# Supplementary material for: ABHD17 proteins are novel protein depalmitoylases that regulate N-Ras palmitate turnover and subcellular localization
Source: eLife. 2015 Dec 23;4:e11306. doi: 10.7554/eLife.11306 (PMC4755737; doi:10.7554/eLife.11306)
Supplement: Supplementary File 3. — A table listing gene-specific primer pairs for verification of transcript levels in HEK293T cells by RT-qPCR in Figure 5A. DOI: http://dx.doi.org/10.7554/eLife.11306.014 [file elife-11306-supp3.docx]

**Supplementary file 3.** List of gene-specific RT-qPCR primer pairs used in this study.

| **#** | **Gene** | **Oligo Pair Sequences** |
| --- | --- | --- |
| **1** | **β-actin**  **(REFERENCE GENE)** | 5'- ACCGAGCGCGGCTACAG -3'  5'- CTTAATGTCACGCACGATTTCC -3' |
| **2** | **ABHD17A** | 5'-TGCCGACTTCCAGTACAGC-3'  5'-ACATACATGCAGGAGACGCG-3' |
| **3** | **ABHD17B** | 5'-CTGCTCGATATGAGAGTGCTG-3'  5'-GTCAATGTTTGGGAATGCATC-3' |
| **4** | **ABHD17C** | 5'-GTCAGAGCATTGGGACTG-3'  5'-GATATCTTGTCAATGCTGGG-3' |
